# 

**Published:** 1917-10-06

**Authors:** 


### CONTENTS.

| | PAGE |
|---|---|
| **Generalism and Specialism** ... ... ... ... ... | 1 |
| **Medical Controversies**... ... ... ... ... | 2 |
| **Hospital and Institutional News** ... ... ... | 3 |
| The Shortage in the Medical Profession—The New Imperial Order—The Aural Board—The Working of the Venereal Diseases Act, 1917—Average Cost per Bed under the Red Cross—Dr. Marcus Paterson—Poor-Law Infirmaries and Soldiers' Wives—An Uncomfortable Hospital—Filming a Colliery "Accident"—Increased Accommodation for Pathological Work—A Medical Eye-witness on the East African Campaign—Medical Draughtsmen—The Cavell Papers—The Arguments of the Prosecution—What the Defence would have been—"Patriotism is not Enough"—A Superman at the Metropolitan Hospital—This Week's Drug Market. | |
| **Unqualified Medical Practice** ... ... ... | 7 |
| Should it be Suppressed by Law? | |
| **The Institutional Library** ... ... ... ... | 9 |
| **Two Types of Medical Leader** ... ... ... | 11 |
| No. I.—The Hero. | |
| **Duke of Connaught's Auxiliary Hospital, Bray** | 12 |
| For Irish Sailors and Soldiers who have Lost a Limb. | |
| **The Editor's Letter-Box** ... ... ... ... | 13 |
| "A Children's Hospital in Difficulties"—"Refusal to Attend Chapel": The Home's Reply. | |
| **Nursing Affairs in Ireland** ... ... ... ... | 14 |
| The Position of the Smaller Institutions. | |
| **The Matrons' and Sisters' Department** ... | 15 |
| Matrons in Council: IX. Some Criticisms Examined. Needs of the Future. | |
| Round the Hospitals. | |
| The College Nottingham Meeting—The Growth of the Register—Scottish Matrons at Edinburgh—Miss E. Margaret Fox at Ismail—Russian Soldier Patients: Two Aspects—A State-aided Midwifery Service. | |
| **Some Problems of To-day**... ... ... ... | 18 |
| Crèche or Nursery School? | |
| **Training in Massage** ... ... ... ... ... | 19 |
| The Combined Scheme of Two Well-known Hospitals. | |
| **Matrons' and Sisters' Appointments** ... ... | 20 |
| **The Organisation of the Leicester Saturday Society**... ... ... ... ... ... | 20 |
| **The Institutional Worker** ... ... | (Suppl.) 1 |
| Starting a Venereal Block. | |

